# Surface-engineered dual drug-loaded tumor-targeted liposomal nanoparticles to overcome the therapeutic resistance in glioblastoma multiforme

**DOI:** 10.21203/rs.3.rs-6080830/v1

**Published:** 2025-10-31

**Authors:** Ramcharan Singh Angom, Hari Krishnareddy Rachamala, Naga Malleswara Rao Nakka, Vijay Sagar Madamsetty, Paola Saurez Meade, Beatriz I Fernandez Gil, Tanmay Kulkarni, Raegan Weil, Shamit Dutta, Enfeng Wang, Santanu Bhattacharya, Krishnendu Pal, Alfredo Quinones Hinojosa, Debabrata Mukhopadhyay

**Affiliations:** 1 Department of Biochemistry and Molecular Biology, Mayo Clinic College of Medicine and Sciences, Jacksonville, FL 32224; 2 Department of Neurosurgery, Mayo Clinic College of Medicine and Sciences, Jacksonville, FL 32224; 3 Department of Bioengineering, Mayo Clinic College of Medicine and Sciences, Jacksonville, FL 32224

**Keywords:** Glioblastoma, Liposomal nanoparticle, Radiation, Temozolomide, Drug resistance, Targeted therapy, Combination therapy

## Abstract

**Background:**

Glioblastoma (GBM) is the most common high-grade primary malignant brain tumor, characterized by a notably poor prognosis. Current treatments for GBM have shown limited effectiveness in improving patient survival, highlighting the urgent need for novel therapeutic strategies. Combination therapy offers significant potential in overcoming resistance by targeting multiple signaling pathways; however, it often comes with increased toxicity compared to monotherapy.

**Methods:**

We utilized a tumor-targeted liposomal nanoformulation (TTL) and loaded it with everolimus (TTL-E), vinorelbine (TTL-V), rapamycin (TTL-R), a combination (TTL-EV), or (TTL-RV). These formulations were tested in vivo on orthotopic GBM mice, combined with temozolomide and radiation. RNA sequencing was performed to identify molecular and transcriptome changes post-treatment.

**Results:**

TTL demonstrated tumor-specific uptake, effectively delivering drugs to GBM tumors. Radiation combined with TTL-EV/RV improved tumor growth inhibition and survival. Transcriptome analysis revealed differentially expressed genes (DEGs) and pathways associated with immune response, DNA damage repair, cell cycle, metabolism, and extracellular matrix pathways.

**Conclusion:**

TTL crossed the blood-brain barrier, effectively targeting tumors. Radiation plus TTL-EV/RV enhanced tumor suppression and survival in GBM models. Mechanistic studies suggest TTL-EV plus radiation inhibits immune and DNA damage pathways and sensitizes tumors to radiation. These findings offer a potential approach for improving GBM treatment.

## Introduction

Glioblastoma (GBM), the most common and aggressive form of primary brain tumor, presents a formidable challenge in therapeutic intervention. Despite significant advancements in medical science, the management of GBM remains highly challenging due to its invasive nature, heterogeneity, and resistance to conventional treatments^1^. Current therapeutic strategies typically involve a combination of surgery, radiation therapy, and chemotherapy, often with the alkylating agent temozolomide (TMZ). However, even with aggressive treatment, the prognosis for GBM patients remains poor, with a median survival of around 15 months from diagnosis^2, 3^. Moreover, the blood-brain barrier limits the therapy for GBM by preventing many chemotherapeutic agents from effectively reaching the tumor site^4^. As a result, many potential treatments for GBM are either rendered ineffective or require alternative delivery methods to bypass or disrupt the blood-brain barrier, complicating therapeutic strategies^5^.

Combination techniques targeting several signaling pathways or signaling cascade nodes are urgently needed to lessen side effects and boost anti-tumor responses because TMZ can cause unintended systemic toxicity^6^. Numerous clinical investigations demonstrating synergistic effects greater than the total therapeutic outcomes of each medicine have clearly shown the advantages of this approach^7, 8, 9^.

Nanocarrier-based drug delivery systems have recently attracted significant attention due to their biosafety, sustained drug release, enhanced bioactivity, and ability to penetrate the BBB. Specifically, Liposomes are a promising drug delivery platform due to their unique properties. Importantly, their capacity to encapsulate lipophilic and hydrophilic substances enables the incorporation of a wide range of medications with varying solubilities^10^. Moreover, liposomes shield medications from chemical inactivation, enzymatic degradation, and immunological neutralization, ensuring their efficacy until they reach target tissues. Consequently, formulations with liposomal medications often result in significantly fewer adverse drug reactions^11^. Additionally, liposomes boast good biocompatibility and low immunological reactivity, further contributing to their appeal^12^. Numerous liposomal medication formulations, including Doxil^®^, DaunoXome^®^, Depocyt^®^, Myocet^®^, Mepact^®^, Marqibo^®^, and Onivyde^®^, are currently in clinical use^13^. However, it’s worth noting that no FDA-approved liposomal product employs active targeting via a targeting ligand.

Our study utilizes a liposomal nanoparticle formulation to deliver first, everolimus and vinorelbine in GBM xenografts enriched with a novel tumor-targeted peptide (TTP). Next, we tested our liposomal formulation with rapamycin and vinorelbine. We selected this combination based on our prior research demonstrating the enhanced therapeutic efficacy of vinorelbine in Renal Cell Cancer (RCC) when combined with antiangiogenic therapy using a VEGF-neutralizing antibody^14^. Considering the properties of these drugs, we anticipate that the combination of everolimus or rapamycin and vinorelbine will synergistically enhance the effectiveness of vinorelbine, leveraging everolimus’s metabolic regulatory capabilities as shown before in other cancers^14, 15^. Our experimental results demonstrate the exceptional tumor-targeting efficacy of TTP-liposomes (TTL) in GBM cells. We also assess how well TTL-EV or TTL-RV inhibits tumor growth and proliferation *in vitro* using four different GBM cell lines while promoting survival in vivo in *a*n orthotropic mouse model. Transcriptome analysis of the mouse tumor revealed top differentially expressed genes (DEGs) and the pathways affected in the TTL-EV and TTL-EV plus radiation-treated mice compared to the untreated mice. DNA damage and Immune-related pathways were among the top enriched pathways, suggesting an enhanced immune function in these mice. We also identified DEGs that might be potential therapeutic targets in GBM. This study endeavors to advance the treatment landscape for GBM patients by evaluating a dual drug-loaded tumor-targeted liposomal nanoformulation in GBM xenografts, aiming to eventually improve patient outcomes and quality of life.

## Results

### Physicochemical characterizations and drug loading efficiencies of the tumor-targeted liposomal nanoparticles (TTL NPs):

**Figure 1.**
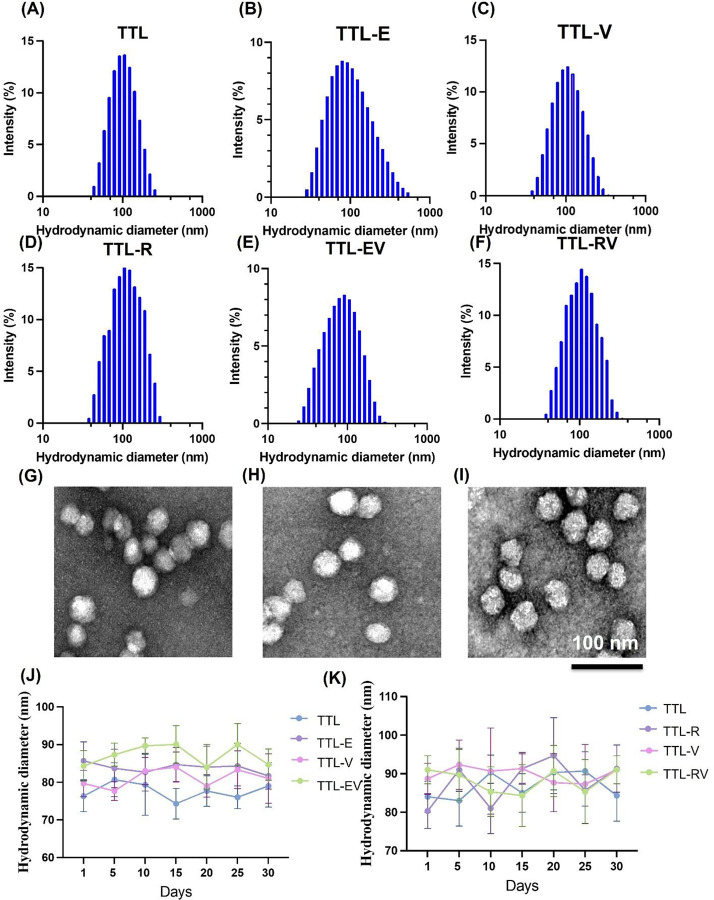
Characterization of dual drug-loaded targeted liposomal nanoformulations. Hydrodynamic size, polydispersity index (PDI) and zeta potential was characterized DLS. **(A)** Empty liposomes (TTL), **(B)** TTL-E, **(C)** TTL-V, (D) TTL-R, (E) combination of Everolimus and Vinorelbine (TTL-EV) and **(F)** combination of Rapamycin and Vinorelbine (TTL-RV). The morphology of transmission electron micrographs with a scale bar of 100 nm was examined **(G)** TTL, **(H)** (TTL_EV, and **(I)** TTL-RV. **(J-K)** Stability of the empty liposome and mono and dual drug-loaded targeted liposomal nanoparticles. All the measurements were performed in deionized water at 25 °C.

The TTL was prepared using a modified ethanol injection method described previously^15^. The physical and chemical properties of liposomes containing Everolimus (TTL-E), Vinorelbine (TTL-V), a combination of both (TTL-EV), Rapamycin (TTL-R), Vinorelbine (TTL-V), and combination (TTL-RV) and empty liposomes (CL) were analyzed. The liposomes’ size was found to range from 70 to 90 nm in diameter and displayed positive zeta potentials between 15 and 21mV (Fig 1A–F and Table S1 and S2), which is in agreement as described in our previous paper^15^. The polydispersity index (PDI) values were between 0.1 to 0.2 for all the TTL NPs (Table S1 and S2). All the LNPs formulations were uniformly distributed and spherical in morphology as confirmed by TEM (Fig 1G–I). Both the LNPs formulations were found to be stable up to 30 days (Fig 1J and K).

### The tumor-targeted L-NPs (TTL) displayed enhanced cellular uptake in GBM cell lines

**Figure 2.**
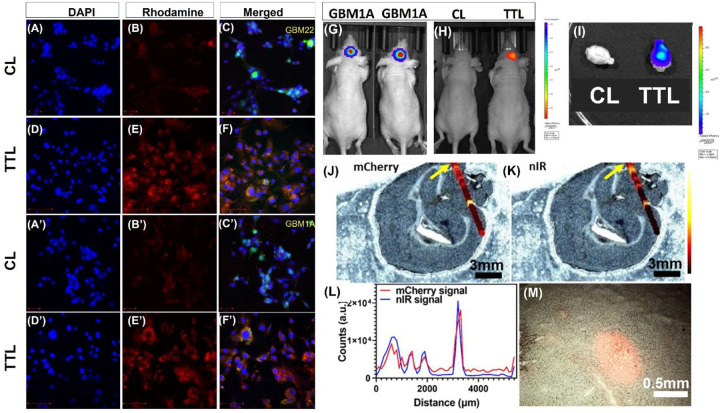
In vitro and In vivo Tumor Uptake Assay: **(A-C)** Representative confocal image showing the uptake of rhodamine-conjugated liposome with and without tumor targeted peptides in GBM 22 cells and GBM 1Z cell line. **(A-C)** Control liposome, (CL) **(D-F)** tumor targeted liposome (TTL). **(A’-C’)** CL, **(D’-F’)** targeted nanoparticles. GBM 1Z and GBM 22 cells were treated with rhodamine conjugated liposome (CL) or TTL for 30 min. DAPI-labeled nuclei are shown in blue and rhodamine in red. The merged image shows GFP luciferase labelled GBM cells in green along with DAPI and the Rhodamine. Images were captured by LSM 800 confocal fluorescence microscopy using blue (405), green (488) and red channels. **(G)** Bioluminescence (IVIS) image of GBM 1Z-orthotropic nude mice. **(H)** Nude mice showing nIR signal in the brain showing tumor specific TLL expression. **(I)** Bioluminescence (IVIS) image of dissected GBM 1Z-orthotropic mice Brain. Consistent colocalization of mCherry and nIR signals confirming tumor specific accumulation of tumor targeted liposome. Representative line scan containing 55 data points on a mouse brain section using Renishaw Raman-PL microscope **(J)** mCherry, **(K)** nIR signal from the same region of interest. Color bar represents variation in signal intensity from 0 to 2.5×104 counts. Scale bar: 3 mm. **(L)** Spatial distribution of mCherry and nIR signal intensities at the line scan, which starts from the arrowhead in Fig (J). and (K). **(M)** Optical image of GBM tumor using Axio observer. Z1 from Zeiss; red represent the signal from mCherry. (Scale bar: 0.5 mm). n=3 images were analyzed. Bar Length in the confocal image = 100 μm.

We analyzed Rhodamine-PE-labeled TTL for *in vitro* cellular uptake. As can be seen from Fig 2, after 30 min treatment, cellular uptake of the TTL was considerably higher in both GBM 22 (Fig 2A–F) and GBM 1Z (Fig 2A’–F’) cell lines than that of control liposome (CL) prepared using the same proportion of lipids except TTL. This demonstrates the excellent targeting efficiency of TTL.

### In vivo biodistribution of the TT-liposomes in GBM xenograft-bearing mice

Next, we analyzed the *in vivo* biodistribution of the liposomes in GBM tumor-bearing mice. We developed orthotropic GBM 1Z tumors in male nude mice using luciferase-expressing GBM cells and injected nIR-780-dye labeled liposomal nanoparticles via an intravenous route (Fig 2G). We used nIR-780-dye in this experiment as it absorbs and emits less absorbed by live tissue in the nIR region of the spectrum. As demonstrated in (Fig 2G–I and S1), TTL showed a higher tumor-specific signal than CL in the xenografts 24 hrs. after administration. Further, as shown in (Fig 2J–K), we confirmed the TTL localization by using Raman spectrophotometry and mCherry-expressing GBM cells. We performed Renishaw’s “line scan” method to select the region of interest (ROI) as indicated in the grayscale images in Fig 2J and 2K. Peak spectral acquisition for the mCherry signal was observed at 604 nm from the tumor cells and 804 nm for the nIR signal for tumor-targeted liposome (TTL) indicated in the color bar. Fig 2L shows the quantification of the fluorescence (mCherry; red curve) and nIR (blue curve) signal intensity at each data point. Fig 2L shows a consistent colocalization of mCherry and nIR signals confirming tumor-specific accumulation of nanoformulations and the ability of the TTL to penetrate the blood-brain barrier, thus targeting the tumor site. Further, we spotted cherry-labeled GBM 1A cells in the tissue section using a Zeiss Axio observer. Z1 at a higher magnification under a fluorescent filter (Fig 2M).

### Dual drug loaded TTL combination with TMZ or Radiation enhances the cytotoxicity in GBM cells

We tested the dual drug-loaded liposomal nanoparticle formulations for their effectiveness in reducing in vitro cell viability in GBM 22 and GBM 1Z cells. To our knowledge, TMZ and radiation are the standard treatments for GBM. We assessed the cytotoxicity of individually drug-laden liposomal preparations for their efficacy in reducing in vitro cell viability in two different cell lines GBM 22, and GBM 1Z cells. These two temozolomide-resistant cell lines were treated with temozolomide at different concentrations, ranging from 10 μM to 70 μM, and we found no toxicity in either cell line (Fig S2A and Table 1). In the GBM 22 cells, IC_50_ of TTL-EV formulations with respective E and V were observed in the concentrations at 0.452 μM 0.272 μM, respectively. A combination of TMZ showed an additive cytotoxic effect (Fig S2A). For GBM 1Z cells, we observed the IC_50_ concentrations at 0.122 μM for everolimus and 0.073 μM for vinorelbine in TMZ, and the TTL-EV combined showed an additive cytotoxic effect (Fig S2B). Thus, the dual drug-loaded liposomes had better efficacy in the GBM 22 (Fig S2A) and GBM 1Z (Fig 2B) cell lines.

We then selected another standard treatment modality for GBM (Radiation). The GBM cells were treated with 2Gy radiation for 30s. before starting the TTL-EV treatment. We observed that individual formulations such as TTL-E, TTL-V, and their combination formulation (TTL-EV) showed higher cytotoxicity along with the radiation in both cell lines (Fig S2C-F and Table 2). Notably, we observed that the dual drug-loaded liposomes (TTL-EV) had better efficacy in all the cells tested GBM 22 (Fig S2C and D) and GBM 1Z (Fig S2E and F). The difference between TTL-V and TTL-EV in both cell lines was minimal without radiation (Fig S2C and E). The above results demonstrated that dual drug-loaded liposomes efficiently decreased cell viability, and the combination, either with TMZ or radiation, further enhances their cytotoxic effect. Interestingly, we observed better cell viability inhibition upon TTL-EV and radiation combination treatment, suggesting this combination to be a promising therapeutic approach. To further demonstrate the effectiveness in other cell lines, we performed similar experiments on two more patient-derived GBM cell lines, QNS120 and QNS108. We observed a significant reduction in the IC50 of both cell lines (Fig S3 and Table S3).

### TTL-EV and Radiation inhibit colony formation and migration in GBM cells:

**Figure 3.**
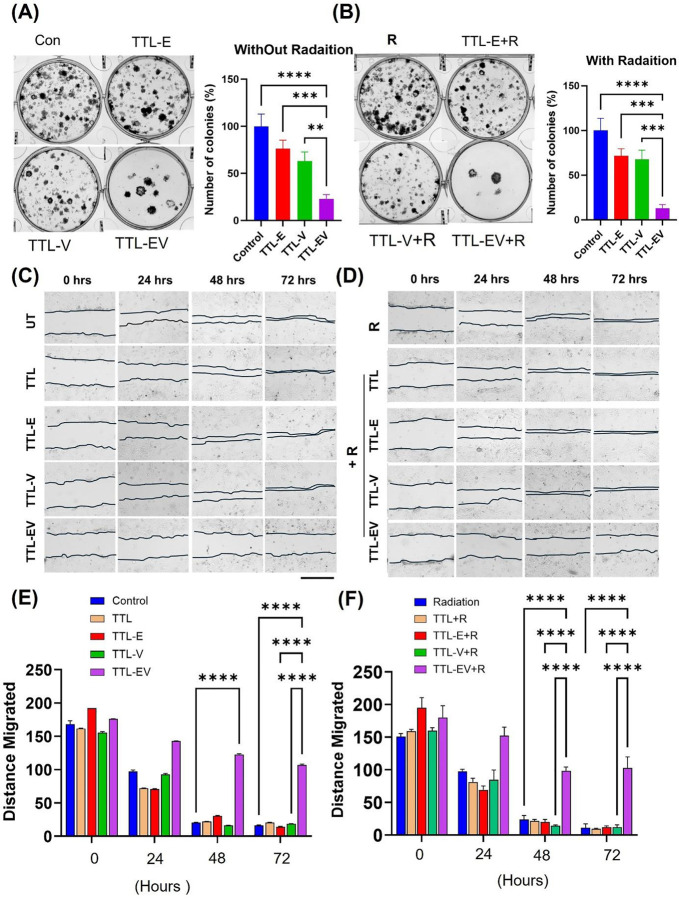
TTL-EV demonstrates an antiproliferative effect and radiosensitization in GBM cells. **(A-B)** Clonogenic assay in GBM 22 cells for determining radiosensitization in vitro. Representative images of the colonies and Colonies greater than 50 cells were counted under a microscope and were plotted (**A)**. GBM 22 cells treated with TTL, TTL-E, TTL-V, and TTL-EV without Radiation. (**B)**. GBM 22 cells treated with TTL plus Radiation, TTL-E plus Radiation, TTL-V plus Radiation, and TTL-EV plus Radiation. **(C-D)** Wound healing assay showing GBM 22 cell migration treated with TTL, TTL-E, TTL-V and TTL-EV **(C)** without radiation, **(D)** with radiation. **(E-F)** Quantification of. The bars represent Mean ± SD. ** p<0.01, *** p < 0.001, **** p < 0.0001.

To further understand the drug’s capacity to enhance the ability of radiation therapy to inhibit colony formation, we evaluated the clonogenicity of the GBM 22 cell line after radiation treatment followed by 72 hours of TTL-E, TTL-V, and TTL-EV treatment (0.3μM of E and 0.1 μM of V). The cells were then plated on six-well plates (each well was seeded with 100 cells) and allowed to grow for 14 days. Representative images of the colonies for the GBM 22 cell line and the corresponding quantifications of the results are displayed in (Fig 3A–B). Compared to the untreated (CL) group, TTL-E (70%), TTL-V (65%), and TTL-EV (25%) showed a significantly lower number of colonies. We observe that as compared to untreated with radiation (UT+R), TTL-E + radiation group showed a significantly smaller number of colonies (<70%). TTL-V + radiation (<65%) and TTL-EV + radiation (10%). Next, we analyzed the GBM 22 cell migration using wound healing assay as described before^16^. The GBM 22 cells treated with TTL-EV and TTL-EV plus radiation displayed more significant migration inhibition (Fig 3 C–F).

### TTL-EV combined with radiation inhibits GBM cell motility

**Figure 4.**
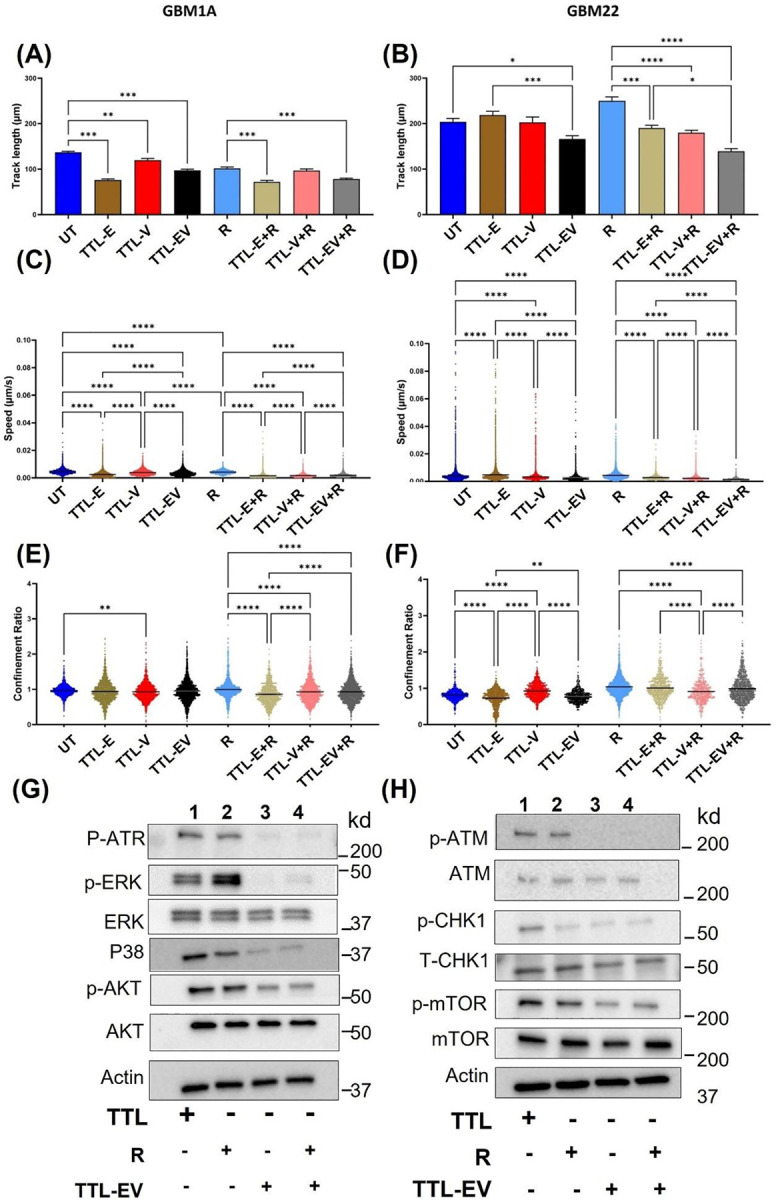
TTL-EV and TTL-EV plus Radiation reduced the cell motility and DNA damage protein expression. Track length measurements reveal that GBM 1Z **(A)** and GBM 22 **(B)** cells treated with TTL-E and TTL-EV show reduced migration distances compared to control cells, irrespective of radiation treatment. Track speed is significantly reduced across all groups compared to their controls for GBM 1Z **(C)** and GBM 22 **(D)**. Non-radiated GBM 1Z cells treated with TTL-V **(E)** and non-radiated GBM 22 cells treated with TTL-E **(F)** exhibit a decreased confinement ratio. **(G)**. Western blot shows DNA damage pathways proteins expression in the treatments. **(G-H)**. Western blot shows tumorigenesis and DNA damage protein expression in the treatments. * p<0.05, ** p<0.01, *** p<0.001, **** p<0.0001. n=3 independent experiments were performed.

Next, we analyzed the effects on motility using the platform Livecyte. We found that the track length (distance; the cells migrate), speed, and confinement ratio are consistently reduced in both TTL-E and TTL-V treatments for GBM 1A (Fig 4A, C, and E) and GBM 22 (Fig B, D and F). We observed an enhanced effect when combined with radiation compared to the other groups (without radiation). Compared to the untreated group, treatments with TTL-E and TTL-V without radiation also displayed reduced speed, length, and confinement ratio in these cells. Further, as in GBM 1A, the confinement ratio, which indicates the higher meandering rate, is lower for TTL-E and TTL-V combined with radiation (Fig 4F), which implies that the cells meander more. The combination of TTL-EV plus radiation significantly reduced all three parameters tested in both cell lines (Fig 4A–F).

### TTL-EV reduces the expression of tumorigenesis-related proteins in the GBM cells.

To investigate the TTL-EV mediated molecular mechanisms in GBM cell lines, we first tested the effect of TTL-EV in combination with the classical drug TMZ by treating the GBM 1Z cell lines with either TTL-EV, TMZ, or TMZ plus TTL-EV. We examined the expressions of proteins related to tumorigenesis and proliferation (Fig S4 A-B). Inhibition of the AKT pathway is identified as a potential treatment approach in GBM. TMZ + TTL-EV treatment significantly suppresses the phosphorylated AKT in the GBM1 A cell lines (Fig S4A). AKT is downstream of serine/threonine kinase in the RTK/PTEN/PI3K and AKT and phospho-AKT levels are elevated in most GBM due to the mutated RTK/PTEN/PI3K pathway^17^. MAPK family, including ERK and P38 MAPK, are involved in a wide variety of cellular processes, comprising proliferation, differentiation, transcription regulation, and development^18, 19^. Consequently, we explored ERK and P38 MAPK expressions. Our results confirm that ERK and P38 are upregulated in GBM^20^. The TTL-EV combination with TMZ suppresses the expression of P38 and ERK proteins (Fig S3B). We also tested the MGMT and ABCG2 protein expression (Fig S4B). It has been shown that patients whose cancer cells express MGMT do not typically respond to TMZ^21, 22^. We observed suppression in the expression of both MGMT and ABCG2 proteins when the cells were treated with the combination (Fig S4B). Next, we tested the effect of combination treatment with radiation using a similar approach. TTL-EV plus radiation combination treatment suppress protein expression of phospho-ERK, Phospho-AKT, and P38 protein expression (Fig 4G). Notably, our results show that the TTL-EV and radiation suppress the expression of phospho-ATM, phospho-ATR, P-mTOR and phosph-CHEK1 (Fig 4H). This further suggests this combination therapy approach affects the DNA damage response (DDR) pathway in GBM.

### *In vivo* efficacy of dual drug-loaded targeted liposomes in GBM xenografts

To examine the efficacy of the TTL in PDX models we treated the mouse bearing GBM 1Z xenografts with TMZ alone, TTL-EV alone and TTL-EV plus TMZ. Briefly, we selected a standard chemotherapy drug for GBM (TMZ) combined with the dual drug formulation (TTL-EV) and treated in cohorts of 5 mice per group, as shown in the outline (Fig S5). Tumor volume analysis shows tumor inhibition when treated with TTL-EV as compared to control but overall, no significant difference was observed between TTL-EV alone and the TTL-EV plus TMZ combination treatment (Fig S6 A-D). Survival analysis of mice treated with TTL-EV plus TMZ-combination show improved survival compared to untreated and TMZ alone. However, we didn’t see a significant between TTL-EV plus TMZ (41 days) and TMZ alone (39 days) (Fig S6F). Moreover, the histological analysis by Ki67 and CC3 staining demonstrates no significant differences in the antiproliferative activity in these groups (Fig S6G).

**Figure 5.**
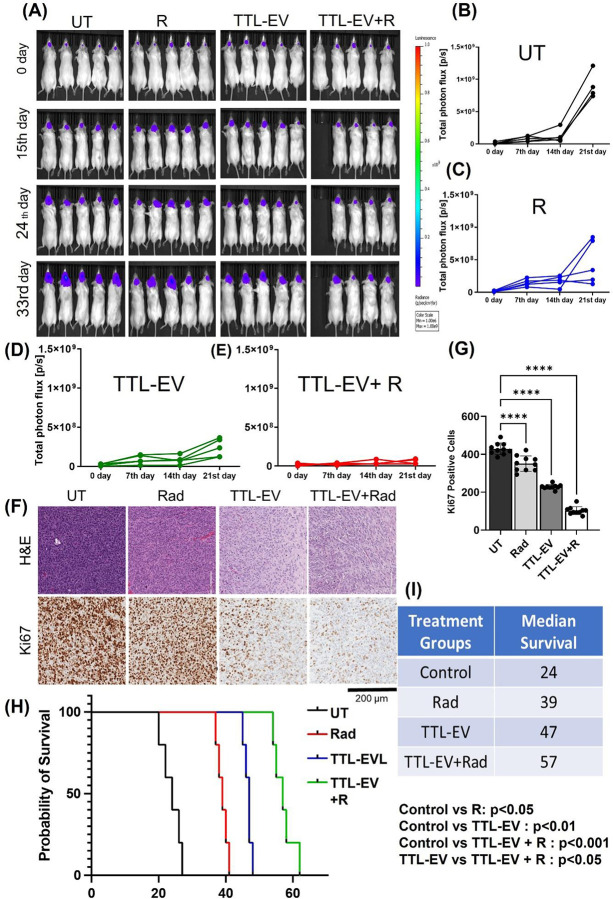
In vivo tumor regression study of TTL-EV, and TTL-EV plus radiation in an orthotopic glioblastoma (GBM) tumor model. **(A)** IVIS image of the GBM mice with different treatments. Luciferase quantification of **(B)** UT **(C)**, Radiation (R) **(D)**, TTL-EV, and **(E)** TTL-EV plus radiation. **(F)** Histological analysis of the tumor section shows H and E and Ki67. **(G)** Quantification of Ki67 positive cells in F. **(H)** Kaplan Meier survival plot showing median overall survival from the above experiment and **(I)** Statistical analysis of the medial survivals. * p < 0.05, ** p < 0.01 and *** p<0.001. For the above study, n=5 mice were analyzed in each group.

To test whether this observed effect of TTL-EV and TMZ can be extended to another regimen, we selected the formulation TTL-EV for further validation studies in combination with radiation in cohorts of 5 mice as shown in the outline (Fig S5A). We saw a remarkable decrease in the tumor volume from the starting volume in GBM 1Z xenografts when treated with radiation plus TTL-EV as indicated by the IVIS images (Fig 5A) and the tumor volume analysis (Fig 5B–E). Further, the tumor sections were stained with H&E to analyze the tumor growth (Fig 5F). The tumor sections were subjected to Ki67 staining to analyze the proliferation state of the tumors (Fig 5F). The histological analysis confirms the tumor growth inhibition as indicated by the smaller H&E-stained area (Fig S7A) and suppressed proliferation as indicated by the reduced Ki67 positive staining (Fig 5F, G and S7A) in the tumor treated with the TTL-EV plus radiation combination therapies. We observed a significant improvement in median survival when treated with TTL- EV plus radiation (57 days) as compared to Control (24 days), TTL-EV alone (47 days), and radiation alone (39 days) (Fig 5 H–I). We didn’t observe any adverse effects due to the treatment, as shown by the histological analysis of the organs isolated from the representative mice (Fig S7B). Next, to further validate our results, we tested the efficacy of our TTL with a second drug combination, rapamycin, and vinorelbine (TTL-RV). As observed in case of TTL-EV, we show significant tumor growth inhibition and enhanced median survival (55 days) of the GBM mice when treated with the combination of TTL-RV plus radiation when compared to control (22 days), radiation alone (40 days), and TTL-RV alone (45 days) (Fig S8). Our analysis demonstrates that radiation combined with the dual drug formulation (TTL-EV/RV) exhibited better overall survival and tumor growth inhibition outcomes than the combination of TTL-EV plus TMZ.

### Transcriptomic profiling of GBM tumors reveals differentially expressed genes and enriched pathways

**Figure 6:**
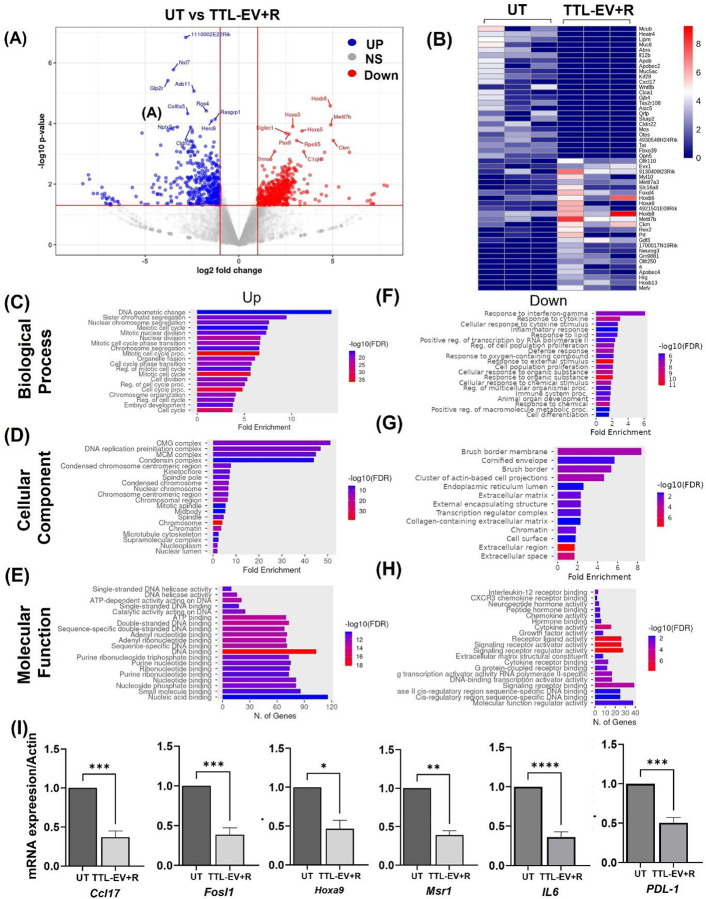
Transcriptome analysis revealed differential gene expression induced by TTL-EV and TTL-EV plus radiation therapy. **(A)** Volcano plot showing the DEGs between control and TTL-EV plus radiation (TTL-EV + R). **(B)** The heat map shows the top-expressed DEGs between controls (UT) and the TTL-EV +R. **(C-E)** Gene ontology analysis: bar plots showing the top 10 enriched ontology terms (GO) analysis was performed using ShinyGO 0.82 to identify enriched biological processes, molecular functions, and cellular components associated with significantly upregulated in TTL-EV + R when compared to UT. **(F-H)** : Bar plots showing the top 10 enriched GO terms associated with significantly downregulated genes between controls (UT) and the TTL-EV + R. **(I)** mRNA expression analysis using qRT PCR..For mRNA analysis, the experiments were repeated three times. The bars represent the mean ± SD. *, p<0.05, **, p<0.01, ***, p<0.001.

To systematically investigate the drug-loaded liposomes and the combination’s biological impact on GBM, we performed transcriptional profiling of the GBM 1Z tumor-bearing mouse brain treated with TTL-EV alone and TTL-EV plus Radiation. First, at the transcriptome level, we identified 76 upregulated genes and 578 down-regulated genes in control vs TTL-EV alone treatment groups, 538 upregulated genes and 443 downregulated in control vs. TTL-EV plus radiation, and 952 upregulated genes and 427 down-regulated genes in the groups treated with TTL-EV alone Vs. TTL-EV plus radiation (Table S4). Top differentially expressed genes in TTL-EV plus radiation tumor were identified and presented in the volcano plot (Fig 6A), and selected top 25 upregulated and 25 downregulated genes were presented on the heat map (Fig 6B). Kyoto Encyclopedia of Genes and Genomes (KEGG), and, Gene Set Enrichment Analysis (GSEA) showed that genes in the pathways involving “cytokine-cytokine interaction, cellular response to interferon-gamma, T cell activation, cytokine response” were enriched in Control Vs. TTL-EV (Fig S9, S10 and S11). Gene Ontology (GO) enrichment analysis revealed that genes related to “mitotic cell cycle phase transition, cell cycle, DNA replication” and adhesion ranked in the top ten positions, with their molecular functions and cellular components closely related to “DNA-replication, Nuclear fission transcription factor binding” and “chromosomal region” respectively in the TTL-EV plus Radiation (Figs 6C–H, S8B). Similar results were obtained in the GO ORA and KEGG analysis. Further, GSEA indicates that cellular response to interferon-gamma and t-cell activation and cellular response to cytokine stimulus were among the top enriched pathways (Fig 6F). The genes commonly downregulated upon TTL-EV treatment (Log_2_FC >1 and p-value < 0.05) included *Cd3e, Ppp1r17, Fat2, Lhx1, Glra1, Svep1*, *Mab21l2, Serpina1b, Cbln1 Corin, Apol9a, Gbp2, Cybb, Car8, Mab21l1*, and the genes upregulated in this group (Log_2_FC >1 and p-value < 0.05) included, *Obscn, Rps3a2, Prss41, Mettl11b, Rps2-ps10, Scn10a, Rtp1, Has1* etc (Table s4). On the other hand, genes commonly downregulated in TTL-EV plus Radiation treatment included (Log_2_FC >1 and p-value < 0.05) Cell signaling and adhesion (e.g., Col6a5, Ovol2, Wnt10a, Adgrf4), Immune response (e.g., IL12a, CD274, ILR2), Inflammation and Metabolism (e.g., *PTGS2os, Akr1c18, Cd7, Ptgs2*), lipid transport (*APOB*), Stemness (*Ascl2*), junction proteins (*Cldn22, GjB4*) (Fig S7 A-C). Upregulated (Log_2_FC >1 and p-value < 0.05) genes include developmental response to radiation (*Rad54l, Hoxa1, Ect2, Fignl1, Pbk, Ticrr, Chek2*), DNA-binding *HOXB6*, DNA replication (*Eme1, Ticrr, Chaf1b, Rmi2, Cdk1, Mcm2, Cdc45, Rad51, Chek2, Ccna2*, *Stag2*, and development (*GDF3, Hoxb6, Hoxb8*), which regulates stemness. neuronal functional genes (for example, *Foxa2, Hes3, En1, Gsc*), metabolism (*Pax8, Tyms, Ckm, Prdx4, Bbox1, Cpt1b*) activation of innate immune response (*Tlr8, Mapkapk3, Xrcc6, Xrcc5, Nono*). A network analysis revealed major pathways genes regulated in both eh UT vs TTL-EV and TTL-EV+R (Fig S12). The detailed list of DEGs is presented in Tables S6 (UT vs TTL-EV) and S7 (UT vs TTL-EV+R).

### TTL-EV and TTL-EV plus radiation modulate genes linked to radioresistance and immune response in human GBM

Next, to validate their relevance and to provide further insight into how these genes are expressed and their association with clinical outcomes, we performed the expression and survival analysis (Kaplan-Meier) of the DEGs (Log_2_FC >1 and p-value<0.05) in TTL-EV and TTL-EV pus radiation groups. Gene Expression Profiling Interactive Analysis (GEPIA) http://gepia.cancer-pku.cn/ as described in the supplementary method. Based on the result derived from GEPIA, the expression of genes, including *Cd3e, Cd274 (PDL-1)*, *Wt1, Fate, Gbp2*, and *Cybb* were significantly upregulated in the GBM tumor compared to the normal brain. However, the RNA seq of mice GBM brains treated with TTL-EV treatment showed downregulated expression of these genes (Fig S13). Similarly, when analyzed, genes upregulated in the GBM tumors, including *CD7, Ilr2, Cd274(PDL-1)*, *Tmem236, Gbp2, Mcub,* and *Smim5,* were downregulated upon the TTL-EV plus Radiation treatment in GBM mice (Fig S14). Kaplan-Meier analysis showing the correlation with the survival outcome of these genes is presented in Fig S15 and S16.

### qPCR validation confirms the downregulation of key genes identified by transcriptomic analysis

To validate the RNA sequencing results, quantitative PCR (qPCR) was performed to assess the expression levels of selected genes that were found to be significantly downregulated in the TTL-EV+R treated samples compared to controls (UT). The genes analyzed included *Fosl1, Hoxa9, Cxcl10, IL6, CCL17, Msr1*, and *PD-L1*. The qPCR results corroborated the transcriptomic findings, demonstrating significant downregulation of all selected genes in the treated samples (Fig 6I). Pro-inflammatory cytokines and chemokines, including *IL6, Cxcl10,* and *Ccl17*, exhibited substantial reductions in expression following treatment. IL6 is known to promote tumor proliferation and survival in glioblastoma through the JAK/STAT3 signaling pathway^23^. Similarly, Cxcl10 has been associated with glioblastoma progression by enhancing immune cell recruitment and supporting a pro-tumorigenic microenvironment^24^. The observed downregulation of *Ccl17*, which is involved in immune cell trafficking, suggests a diminished inflammatory response that could potentially alter the tumor microenvironment^25^.

A significant decrease in *PD-L1* expression was also observed. PD-L1 is a well-known immune checkpoint protein that contributes to immune evasion by inhibiting T-cell activation^26^. Its downregulation implies that the treatment could enhance immune surveillance by reducing the tumor’s ability to evade the host immune response. Transcription factors such as Fosl1 and Hoxa9 also showed significant downregulation. *Fosl1* plays a critical role in regulating glioblastoma cell proliferation, epithelial-mesenchymal transition (EMT), and invasion through the AP-1 signaling pathway^27^. The reduction in Fosl1 expression suggests a possible inhibition of tumor growth and invasive potential. Similarly, *Hoxa9*, a homeobox gene associated with poor prognosis in various cancers, has been implicated in promoting tumorigenesis through stemness and differentiation pathways^28^. *Msr1 (macrophage scavenger receptor 1)*, which is involved in innate immune response and macrophage activation, also showed reduced expression. Previous studies have highlighted the role of *Msr1* in facilitating tumor-associated macrophage recruitment, contributing to an immunosuppressive microenvironment in glioblastoma^29^. Its downregulation could reflect a shift toward a less immunosuppressive tumor environment. The qPCR validation effectively confirmed the downregulation of key genes involved in inflammation, immune evasion, proliferation, and tumor progression, as initially indicated by transcriptomic analysis. These results suggest that the treatment not only suppresses inflammatory and immune evasion pathways but may also hinder glioblastoma cell growth and invasion.

## Discussion

The glioblastoma (GBM) therapeutic approach involves sequentially administering radiation and chemotherapy following surgical intervention. Notwithstanding the administration of intensive therapeutic interventions, patients’ average overall survival duration remains less than 18 months. Everolimus, also known as RAD-001 or Afinitor^®^, is a pharmacologically accessible compound that inhibits the mammalian target of rapamycin (mTOR). mTOR is a protein kinase crucial in maintaining metabolic homeostasis and is associated with various illnesses, including cancer^30^. Administering everolimus before the Stupp protocol in newly diagnosed patients does not provide any clinical benefit compared to the standard protocol^31^. Combining everolimus with conventional chemoradiation had shown moderate toxicity and did not render an extensive survival advantage^31^. Another phase II study showed that Individuals with recurrent/progressive NF1-associated low-grade gliomas (LGG) exhibit substantial effects during therapy with oral everolimus with a well-endured toxicity profile. Hence, everolimus could still be favorably suitable for future options as upfront or combination therapy in GBM^32^. Further, a recent phase II study has shown that daily oral everolimus provides a well-tolerated, alternative treatment for multiple recurrent, radiographically progressive pediatric LGG. Based on these results, everolimus is further investigated for this patient population^33^.

On the other hand, vinorelbine is used as an anticancer agent in various combination chemotherapy regimens^34^. A case study showed that vinorelbine is effective against high-grade pediatric glioma, and intravenous vinorelbine administration resulted in more than 24 months of disease-free progression^35^. Another report in pediatric high-grade gliomas showed that a combination treatment of vinorelbine and valproic acid added to temozolomide resulted in an improved outcome in the 5-year OS and the 5-year PFS^36^. Vinorelbine also has shown relapsing GBM in 19-year-old female patients diagnosed with GBM located in the deep temporal region^35^. Vinorelbine has been extensively studied both in vitro and in vivo. Its efficacy has been experimentally demonstrated for the treatment of malignant brain glioma. Despite this advancement, the tumor persisted after a rigorous treatment regimen consisting of pre-radiation chemotherapy. We demonstrated that combined TTL-EV/RV plus radiation inhibits the GBM tumor progression and improves survival in the GBM mouse model. Notably, our TTL successfully crossed the BBB and specifically targeted the tumor. Thus, co-encapsulating multiple therapeutic agents into a tumor-targeted drug delivery platform holds promise for overcoming these therapy limitations and improving treatment outcomes.

MAPK ERK cascade has been profoundly investigated as one of the most important signaling pathways in GBM progression^37^. ERK(P44/42) and P38 MAPK regulate various biochemical signals and are concerned with cellular processes, including proliferation, differentiation, transcription regulation, and development. Higher expression levels of ERK and P38 protein have been reported in GBM, and suppression of ERK inhibits GBM growth^20^. Further, as shown previously in GBM, downregulation of phosphorylated ERK upon the treatment was accompanied by reduced protein level of phosphorylated mTOR level, validating the positive correlation between ERK and mTOR pathway. MAPK ERK (P44/42) plays a critical role in GBM proliferation and survival^27^. Previous studies have shown that co-treatment with the ERK inhibitor and mTOR inhibitor rapamycin increased cell mortality and exhibited enhanced antitumor effects on GBM cells^20^. Our results suggest that mTOR and ERK inhibition via TTL-EV rendered GBM tumors radiosensitive in agreement with previous studies^38^.

The PI3K/AKT/mTOR signaling is hyperstimulated in glioblastoma^38^. Studies have shown that activated AKT helps glioma cells grow hysterically, escape apoptosis, and boost tumor invasion, compelling inhibition of AKT an attractive target for GBM therapy. Numerous groups of AKT inhibitors exist. However, few have been tested adequately to show in vivo efficacy. Our results showed that TTL-EV combined with either TMZ or TMZ plus radiation inhibits the expression of phosphorylated AKT protein. Further, low MGMT protein or gene expression is significantly associated with improved patient survival or treatment response independently of MGMT promoter methylation and has also been found to be an independent prognostic marker in glioblastoma patients by multivariate analysis^39, 40, 41, 42^. GBM patients with high expression of ABCG2 (ATP-binding cassette sub-family G member 2, also known as BCRP (Breast Cancer Resistance Protein), proteins activities mediate therapy resistance and apoptosis resistance, respectively^43, 44, 45^. The mRNAs of these two genes are expressed at higher levels in CD133+ GBM cells. Its high protein level had worse survival than those with low expression, and the inhibition of ABCG2 has been implicated as a promising therapeutic target in GBM^46^. Hence, the reduced MGMT and ABCG2 protein expression in the GBM cells treated with TTL-EV again suggested that these dual drug combinations could be a potential therapy avenue in chemo-resistant GBM. Several DDR proteins, including ATM, ATR, and CHK1, are upregulated in GBM stem-like cells, rendering radiation resistance^47^ TTL-EV combined with radiation showed suppression of phosphorylated ATM, ATR, and CHEK1 protein expression, suggesting that it modulates the DDR signaling that mediates treatment resistance by promoting cell cycle arrest in GBM.

In GBM, cell motility is essential for infiltrating adjacent brain areas, whereas distant metastases are rare. Infiltration poses a severe problem for therapy as infiltrated areas cannot be entirely removed by surgery without destroying critical brain structures. As reported earlier^48^ The speed and distance reduction observed in this study will help investigate the specific changes in cytoskeleton reorganization involved in the mechanism of TTL-EV action in GBM cells.

Transcriptome profiling revealed that treatment with TTL-EV and TTL-EV plus radiation in GBM tumor-bearing mice enriched key differentially expressed genes (DEGs) involved in pathways related to immune response, Cell cycle, and DNA damage repair. Notably, markers associated with stemness, cell-cell junction integrity, epithelial-to-mesenchymal transition (EMT), DNA damage repair, adhesion, and migration were significantly downregulated following TTL-EV treatment. Furthermore, critical radioresistance and maintenance genes, including *Cd274 (PD-L1)*, play a role in immune checkpoint regulation and influence the post-radiation tumor microenvironment^49^. Higher expression of PD-L1 is linked with worse outcomes and is a negative prognosticator for GBM outcomes in glioma^50^. According to pertinent research, PD-L1 expression in gliomas is correlated with WHO grade and may serve as a tumor biomarker. PD-1/PD-L1 axis-targeting monoclonal antibodies be safe and effective in preclinical GBM mouse models. Longer animal lifetimes and a notable decrease in tumor size were among the positive outcomes. Clinical trials involving individuals with recurrent glioblastoma multiforme use monoclonal antibodies that inhibit PD-1 and PD-L1^51^. Upregulated PD-L1 binds to PD-1 on T cells, inhibiting their activation and cytotoxic function. This immune suppression allows tumor cells to evade immune-mediated killing despite radiation-induced immunogenic cell death^52^. Wilm’s tumor (Wt1), which is involved in radiation sensitization, apoptosis, and DNA repair mechanism, has been suggested to increase the radiation sensitivity upon its downregulation^53^. Wt1 is a zinc finger transcription factor that plays a complex role in proliferation, differentiation, apoptosis, and DNA damage response. Upregulated WT1 enhances the cell’s ability to repair radiation-induced DNA damage, promoting cell survival and radioresistance. Our result from the transcriptome analysis suggests that TTL-EV and TTL-EVR inhibit key radiation response-related genes, including PDL-1 and Wt1 expression. Further evaluation of TCGA GBM dataset showed differential expressions of the identified genes in GBM and their correlation with survival outcome. Our findings highlight the potential of TTL as a novel therapeutic strategy, as demonstrated by the combination of TTL-EV/RV with radiation, particularly in recurrent or refractory GBM cases. Understanding the underlying molecular mechanisms and patient-specific factors will be critical to optimizing this dual drug-loaded TTL formulation for personalized GBM therapy. Continued research is imperative to overcome current clinical challenges and improve outcomes for GBM patients.

## Conclusion

The tumor-targeted liposomal nanoformulation successfully crossed the BBB and specifically targeted the tumor. The TTL demonstrated superior targeting efficacy in GBM xenograft models. When loaded with dual drug combination (everolimus and vinorelbine or rapamycin and vinorelbine) and combined with radiation, this formulation effectively inhibited GBM cell proliferation in vitro and suppressed tumor progression *in vivo*. Mechanistically, our results suggest that TTL-EV treatment inhibits the mTOR and MAPK pathway, and TTL-EVR treatment sensitizes GBM to radiation and inhibits DNA damage repair. These findings open new avenues for research and identify potential targets to overcome therapy resistance in GBM. Our findings highlight the potential of this tumor-targeted liposomal nanoparticle delivery system, encapsulating everolimus/rapamycin and vinorelbine, as a promising strategy for GBM treatment, addressing the urgent need for more effective therapeutic options. We believe that further validation of these genes will identify a panel of novel biomarkers with significant translational potential contributing to mitigating and improving the clinical management of GBM.

## Materials and methods

### Ethics Statement:

All mice experiments followed the Association for Assessment and Accreditation of Laboratory Animal Care (AAALAC) guidelines under protocols approved by the Mayo Clinic Institutional Animal Care and Use Committee (IACUC) protocol no. A00003448.

### Reagents

Everolimus was purchased from LC laboratories. Vinorelbine was purchased from Fisher Scientific. Antibodies; Anti-Ki67 (ab254123), was purchased from Abcam. Anti-β-actin (A5316 Sigma, USA) was purchased from Sigma. Anti-ABCG2 (cat# ab3380), MGMT (Cat# Mab16200), Antibodies against N-cad (Anti-N-Cadherin, #4061), Anti-mTOR #2972), phospho-mTOR (Rabbit mAb #5536), Anti-EGFR (Rabbit mAb #4267), and Anti-ATM (Rabbit mAb #2873), were obtained from Cell Signaling Technology. Anti-ATR (sc-515173), Anti-Chk1(sc-56288), and Anti-Chk2(sc-17747) antibodies were obtained from Santa Cruz Biotechnology. DiR780 dye, Cell titer 96 Aqueous One Solution Cell Proliferation Assay (Promega).

### Cell culture

GBM 1Z, QNS 120, and QNS 108 cells were maintained in GBM stem cell media as described before16 and GBM 22 were maintained in Dulbecco’s modified Eagle mediums (DMEM; Life Technologies) supplemented with 10% fetal bovine serum (FBS; Fisher Scientific) and 1% penicillin-streptomycin (Invitrogen) at 37°C in a humidified atmosphere with 5% CO2 as described previously by our group16. Cultures of 85–90% confluency were used for all the experiments. All the cell lines used were published previously from our group, GBM 1Z and GBM 2216, QNA 120 and QNS 10817.

### Synthesis of TTP-conjugated lipopeptide and preparation of drug-loaded liposomes

In a previous publication, our group described the TTP-conjugated lipopeptide synthesis and preparation of drug-loaded liposomes in detail15. A description of the experimental procedures is detailed in the Supplementary Material and Methods

#### Liposomal formulation preparation method and Physicochemical characterization:

The physical characterization has been described in a previous study15.

#### Analysis of drug loading and encapsulation efficiency

A detailed experimental method to analyze the drug loading and encapsulation efficiency was described in our previous study15.

### In vivo tumor regression experiment

To test the efficacy of the drug-loaded TT NPs, a single mouse trial (SMT) was used to assess the in vivo tumor regression in GBM 1Z xenografts as described previously18–20. Mice with confirmed tumors were treated with the following: CL, TTL-E, TTL-V, and TTL-EV, two times a week via intravenous route. The LNP amount among treatments was kept constant so that the treated mouse gets 20 μg of everolimus and vinorelbine each. Tumors were measured weekly and plotted to obtain a tumor growth curve. All tumor-bearing mice were euthanized with CO2 after the completion of the experiment, and the tumor-bearing brains were removed and prepared for immunochemistry. To validate the results obtained from the SMT and to access the improved efficiency of combination therapy, we repeated the experiment in cohorts of 5 mice per group, combining the most effective treatment group and TMZ or radiation in GBM 1Z tumor-bearing mice.

### Radiation Treatment (RT) in Mice

Tumor irradiation was administered using the X-RAD SmART irradiator (Precision X-ray, North Branford, CT). The mice receiving irradiation were anesthetized under isoflurane 3% fixed on the stereotactic stage using a bite block and immobilized with ear bars. The first mouse was imaged with scout CBCT (60 kVp, 0.3 mA, F1 2.0 mm aluminum filter, 256 images, 200 μm3 voxel size) to locate the irradiation area. A virtual target was placed at a reproducible location in the mouse brain using the burr-hole where the cells were injected as a reference. The mouse was then imaged with a higher-dose and higher-resolution CBCT protocol (40 kVp, 8.0 mA, 2.0 mm Al filter, 256 images, 100 μm3 voxel size), and the target was adjusted. This high-resolution CBCT was repeated in each mouse. RT was performed using a top perpendicular beam with a circular 3mm collimator and an F2 filter (2 mm copper) at a dose rate of 3.3 Gy/min (225kVp, 13mA). RT treatment was performed over 5 consecutive days.

### Statistical methods

The statistical analysis was performed by using GraphPad Prism 8.0 (GraphPad Prism. Inc., USA). The independent-sample t-test and two-way ANOVA were used to test the probability of significant differences between the groups. P < 0.05 or less indicated a significant difference; P values greater than 0.05 were considered not significant (ns)

### In vitro cellular uptake of Tumor targeted liposomes (TTL)

Cellular uptake assay was investigated using Rhodamine-PE-labeled fluorescent liposomes. The liposomal nanoparticle suspensions were prepared by adding Rhodamine-PE (Avani Polar Lipids) to the organic phase. GBM 22 and GBM 1Z cells were cultured on 96 well plates at a density of 1×104 cells/well for 24 hrs at 37°C. Then, the cells were incubated in fluorescent liposomes for 30 mins. A control liposome without targeting peptide (CL) and with targeted peptide Liposme (TTL) was used to determine the targeting efficiency. The nuclei of the cells were counterstained with DAPI for the last 30 mins. After 30 mins of incubation, the cells were rinsed with PBS (pH 7.4) three times and then overlaid with 100 μL PBS. The cells were immediately imaged using blue (Alexa 405, and red (Alexa 594) channels using a confocal microscope (Zeiss LSM 880).

### In vivo biodistribution of liposomes

Six- to eight-week-old male SCID mice were obtained from in-house breeding and housed in the institutional animal facilities. To establish tumor growth in mice, 5×105 GBM 1Z cells were resuspended in 2.5 μL of HBSS and injected orthotopically into the brain as described before 1. The mice were monitored for tumor growth for 6–7 weeks without treatment. Then, either control (CL) or tumor-targeted liposomes (TTL) containing IR-780-Dye were administered intravenously. Mice were imaged using an IVIS imager 24 hrs. after administration.

### In vitro cytotoxicity assay

The cytotoxicity assay was performed in four different GBM cell lines, including GBM 22, GBM 1A, QNS 120, and QNS 108 as described previously1. Nearly 5×103 cells were seeded in the 96-well plates. After 24 hrs., cells were treated with increasing doses of empty liposome TTL or liposomes containing everolimus (TTL-E), vinorelbine (TTL-V), rapamycine (TTL-R), and a combination (TTL-EV/RV) thereof diluted in respective media and incubated for a further 72 hrs. Cell proliferation was analyzed at the end of the incubation using Cell titer 96 Aqueous One Solution Cell Proliferation Assay (Promega) as per the manufacturer’s protocol. Briefly, the media, including the treatments, were removed by aspiration from the plate and washed with PBS for one time. Then, 100 μL media containing 20 μL One Solution reagent was added to each well. The plate was incubated at 37°C for 30 mins, and absorbance at 492 nm was measured using a SpectraMax i3x instrument. Percentage viability is calculated as follows:

Viability(%)=100×(ATreated−ABlank)/(AUntreated−ABlank).

### Colony formation assay:

To determine the colony formation ability of GBM 22 cells after radiation therapy followed by TTL-EV treatment, GBM 22 cells were seeded into two 6 well plates. One plate was exposed to 2 Gy radiation at room temperature with a 3.9 Gy/min dose rate and a 160 kV tube voltage using an X-RAD 160 Irradiator (Precision X-Ray Inc., USA). After irradiation, the cell samples were returned to a 5% CO2 incubator for four hours. The IC50 concentration of the formulations was selected based on the results from the MTS assay. Then, both plates were treated with different formulations, including CL (Control liposomes), TTL-E, TTL-V, and TTL-EV, for 72 hours. After treatment, irradiated and non-irradiated cells were harvested and seeded in triplicates (100 cells/well) in 6-well plates in fresh culture media without antibiotics. The cells were allowed to grow for 16–18 days, and then colonies larger than 50 μm in diameter were counted after fixing them with 4% formaldehyde and stained with 0.2% Crystal Violet solution2.

### Cell Migration Assay

To analyze the cell migration, GBM 1Z, and GBM 22 cells were seeded in a glass-bottom 96-well plate (Cellvis, P96–1.5H-N) at a density of 8 × 103 cells per well in six replicates. After 24 h, cells were irradiated at 2 Gy (X-Rad160, Precision X-Ray), and 6 hours later, cells were treated with TTL-E, TTL-V, and TTL-EV. The time-lapse images were acquired for 3 days in 30-minute intervals using a 10X objective at 37 °C, 5% CO2, and 95% humidity using Livecyte^™^ (Phase Focus Limited), a label-free high-content microscopy. Data were analyzed using the Livecyte^™^ integrated analysis suite (Phase Focus Limited)3.

### Wound Healing Assay

To further analyze the effect of TTL-EV and TTL-EV plus radiation on cell migration, GBM 22 cells were seeded in a 6-well plate at a density of 200 × 10^3^ cells per well in three replicates. After 24 h, cells were irradiated at 2 Gy (X-Rad160, Precision X-Ray), and 6 hours later, cells were treated with TTL-E, TTL-V, and TTL-EV. The cell monolayer was scrapped in a straight line to create a “scratch” with a p200 pipet tip. The debris was then removed washing once with 1 ml of the growth medium and then replaced with 2 ml of medium4.The images were acquired at 24h, 48h, and 72 h using a 4X objective using EVOS microscope (Molecular Probe). Data were analyzed using the ImageJ software5.

### Fluorescence microscopy:

The fluorescence spectra were acquired using the Renishaw InVia Raman Microscope system and set to confocal mode. We performed a “line scan” on the region of interest (ROI) using 10s exposure time and a 5X objective lens while using the Renishaw Raman-PL imaging system. To restrict the tissue burning, we have used a 532 nm laser with 1% laser power and spot size 2.82 μm for mCherry and a 785 nm laser with 0.5% laser power and spot size 4.02 μm for nIR dye. Additionally, we used 2400 I/mm and 1200 I/mm grating for mCherry and nIR dye, respectively. The spectral acquisition was performed at 604 nm for the mCherry signal and 804 nm for the nIR signal. The number of accumulations incorporated was 7 to capture mCherry and nIR signals. Upon acquisition of the fluorescence signal, it was baseline corrected using Renishaw’s WiRE 5.2 data analysis software by employing the “intelligent polynomial” fitting mode of the order 11 and noise tolerance of 1.5 a.u. The raw data was extracted and plotted using Origin Lab software.

### Immunohistochemistry

The control and tumor-bearing mice brains were resected and fixed in 10% formalin at room temperature for at least 24 hrs. before processing for the sectioning. The sections were deparaffinized and then subjected to hematoxylin, eosin (H&E), cleaved caspase 3 and Ki67 immunochemistry as per the manufacturer’s protocol (DAB 150; Millipore). The Histology core facility at Mayo Clinic performed the sectioning and staining. Photographs of the entire cross-section were digitalized using the Aperio AT2 slide scanner (Leica). Images were analyzed using ImageScope software (Leica).

### RNA-seq Profiling and Analysis

As previously described, RNA-seq was performed at the MEDGENOME (USA)6. The mice brain-bearing tumors in the three groups, Control (UT), TTL- EV, and TTL-EV plus Radiation (TTL-EV + R) were dissected, and total RNA was isolated using TRIzol reagent. RNA concentrations were quantified using a NanoDrop Spectrophotometer (Thermo Fisher), and RNA integrity was assessed using the RNA Nano 6000 Assay Kit on a Bioanalyzer 2100 system (Agilent Technologies). Only samples with RIN values of >6.0 were used for experiments. A complementary DNA library was prepared, and sequencing was performed according to the Illumina standard protocol by Beijing Novel Bioinformatics Co., Ltd. (https://en.novogene.com/). Specifically, cDNA libraries were prepared using an Illumina NEBNext UltraTM RNA Library Prep Kit. After cluster generation, the library preparations were sequenced on an Illumina NovaSeq 6000 platform, and 150 bp paired-end reads were generated. For the data analysis, raw data (raw reads) in fastq format were first processed through in-house Perl scripts. Clean reads were obtained by removing reads containing adapters, poly Ns, and low-quality reads from raw data. Reference genome and gene model annotation files were downloaded directly from the genome website. The reference genome index was built using Hisat2 v2.0.5 and paired-end clean reads were aligned to the reference genome using Hisat2 v2.0.5. Mapped reads of each sample were assembled using StringTie (v1.3.3b) in a reference-based approach. Feature Counts v1.5.0-p3 were used to quantify the read numbers mapped to each gene. Differential expression analysis was conducted using DESeq2 (v. 1.38.3). Differentially Expressed Genes (DEGs) with a p-value < 0.05 were visualized as a volcano plot using the ggplot2 package (v. 3.4.2) and the ggrepel package (v. 0.9.3). Heatmaps were generated using the pheatmap package (v.1.0.12). After converting the official gene symbol IDs to Entrez IDs using the org.Hs.eg.db package (v.3.16.0), DEGs were mapped to KEGG (Kyoto Encyclopedia of Genes and Genomes) and GO (Gene Ontology) pathways using the clusterProfiler package (v.4.6.2). For Gene Set Enrichment Analysis (GSEA), each gene’s fold change (FC) between subtypes was first calculated, followed by descending sorting of the input genes based on FC values. GSEA was performed using the xPierGSEA function in the Pi package (v.2.10.0), and related plots, including specific genes showing the leading edge, were drawn using the xGSEA dot plot function. Gene Set Variation Analysis (GSVA) was conducted using the GSVA package (v.1.46.0), and results were presented as a volcano plot. All analyses were performed in R v.4.2.4.

### Western Blotting

Western blot was performed as depicted before1. Briefly, the total cellular protein was extracted with NP40 buffer (Applied biosystem) containing protease and phosphatase inhibitor cocktail (Calbiochem) and was denatured by adding 6x Laemmli sample buffer and heating for 5 min. 10μg of proteins were subjected to SDS-PAGE using 4–20% gradient Tris-glycine gels (BioRad) and transferred to polyvinylidene fluoride (PVDF) membrane. The membrane was blocked in TBS-T buffer having 5% BSA. The primary antibody was diluted in TBS-T having 3% BSA and incubated overnight at 4°C. The horseradish peroxidase (HRP)-conjugated secondary antibody (Cell Signalling Technology) was diluted in 3 % TBS-T and incubated for one hour at room temperature. SuperSignal West Pico Substrate (Thermo Scientific, Rockford) was used to develop the blot.

### Gene expression and survival analysis using GEPIA

Gene expression analysis was conducted using GEPIA (Gene Expression Profiling Interactive Analysis), a web-based tool for exploring RNA sequencing expression data derived from The Cancer Genome Atlas (TCGA) and the Genotype-Tissue Expression (GTEx) projects7. GEPIA was accessed at http://gepia.cancer-pku.cn. The parameters used are described as follows: Cancer type was focused on GBM datasets using matched normal expression data from TCGA and GTEx datasets. The differential Gene expression analysis module of GEPIA was used. The log2 fold change (log2FC) threshold was set at 1.0 (corresponding to a two-fold change in expression). The p-value cutoff was set at 0.01 for statistical significance.

### RNA sample collection and RT-PCR

Total RNA was prepared using the RNAeasy kit (QIAGEN), according to the manufacturer’s protocol. Reverse transcription reaction (RT) was performed to generate cDNA using the IScript^®^ RT-PCR kit (BioRad, USA.). For qPCR, SYBR^®^ Premix Ex Taq^™^ II (Applied biosystem and Life technology.) was used, according to the manufacturer’s instructions, using a 7500 Fast Real-Time PCR system (Applied Biosystems, Foster City, CA, USA). Each 20 μl reaction contained ~500 ng DNA template and 1μM of the forward and reversed primers. The *β-Actin* gene served as an endogenous control. The primer sequences used in the present study are listed in Table S5. Expression levels were normalized to the housekeeping gene *β-Actin*, and relative expression was calculated using the 2^-ΔΔCt method.

## Supplementary Files

This is a list of supplementary files associated with this preprint. Click to download.

- SupplementalTableS6.pdf
- SupplementalTableS7.pdf
- NatCSupplementaryDataFinal.pdf

## Figures and Tables

**Table 1:** IC50 concentrations of the E and V with TMZ

Formulations	GBM 22 (μM)	GBM 1A (μM)
TMZ	>70	>70
TTL-EV+TMZ	0.452±0.002 (E)	0.122±0.004 (E)
	0.272±0.02 (V)	0.073±0.002 (V)
	40.78±0.03 (TMZ)	26.06±0.08 (TMZ)

**Table 2:** IC50 concentrations of the E and V with and without radiation treatment

Formulations	GBM 22		GBM 1A	
	Without Radiation (μM)	With Radiation (μM)	Without Radiation (μM)	With Radiation (μM)
TTL-E	>5	>5	>5	3.237
TTL-V	>3	1.163	2.371	0.6335
TTL-EV	1.4±0.001 (E)	0.3±0.012 (E)	1.05±0.005 (E)	0.193±0.002(E)
	0.85±0.02 (V)	0.178±0.004 (V)	0.63±0.01 (V)	0.116±0.001 (V)

## Data Availability

All the sequencing and transcriptome datasets generated or analyzed in this study will be made available to the research community, and sequences will deposited into a public database like SRA.
